# Injection site microflora in persons with diabetes: why needle reuse is not associated with increased infections?

**DOI:** 10.1111/apm.13230

**Published:** 2022-05-17

**Authors:** Sofia Wareham‐Mathiassen, Lene Bay, Vera Pinto Glenting, Naireen Fatima, Henrik Bengtsson, Thomas Bjarnsholt

**Affiliations:** ^1^ Department of Immunology and Microbiology Copenhagen University Copenhagen Denmark; ^2^ Department of Front‐End Innovation, Devices & Delivery Solutions Novo Nordisk A/S Hillerød Denmark; ^3^ Department of Packaging & Materials Development, Devices & Delivery Solutions Novo Nordisk A/S Hillerød Denmark; ^4^ Department of SaMD Design control & Engineering Novo Nordisk A/S Søborg Denmark; ^5^ Department of Clinical Microbiology Rigshospitalet Copenhagen Denmark

**Keywords:** Injection sites, insulin delivery, medical devices, needle reuse, skin microflora

## Abstract

Needle reuse is a common practice and primary cause of customer compliance issues such as pain, bruising, clogging, injection site reactions (ISR), and associated lipodystrophy. This study aimed to characterize skin microflora at injection sites and establish microbial contamination of used pen injectors and needles. The second objective was to evaluate the risk of infections during typical and repeated subcutaneous injections. 50 participants with diabetes and 50 controls (n = 100) were sampled through tape strips and skin swabs on the abdomen and thigh for skin microflora. Used pen injectors and needles were collected after *in‐home* use and from the hospital after drug administration by health care professionals (HCPs). Samples were analyzed by conventional culture, matrix‐assisted laser desorption/ionization‐time of flight (MALDI‐TOF), mass spectrometry (MS), confocal laser scanning microscopy (CLSM), and 16S/ITS high throughput sequencing (HTS). A mathematical model simulated the risk of needle contamination during injections. Injection site populations were in 10^2^ cells/cm^2^ order, with increased viable bacteria and anaerobic bacteria on the skin in persons with diabetes (p = 0.05). Interpersonal variation dominated other factors such as sex or location. A higher prevalence of *Staphylococcus aureus* on abdominal skin was found in persons with diabetes than control skin (p ≤ 0.05). Most needles and cartridges (95% and 86%) contained no biological signal. The location of the device collection (hospital vs home‐use) and use regimen did not affect contamination. CLSM revealed scarcely populated skin microflora scattered in aggregates, diplo, or single cells. Our mathematical model demonstrated that penetrating bacteria colonies during subcutaneous injection is unlikely. These findings clarify the lack of documented skin infections from subcutaneous insulin injections in research. Furthermore, these results can motivate the innovation and development of durable, reusable injection systems with pharmacoeconomic value and a simplified and enhanced user experience for patients.

The global prevalence of diabetes is rapidly increasing with severe individual and societal costs. Currently, subcutaneous injections (SCIs) of insulin remain the most common form of treatment in the industrialized world. Administration with a pen injector represents approximately 60% of insulin users globally, with higher percentages in Europe, Japan, China, and Australia (80–95%), but only about 20% of users in United States and India [1]. According to a recent report, the insulin pen injector market size is forecasted to grow at a 10.7% rate from USD 57 million in 2021 to USD 117 million by 2026 [2]. While currently‐marketed needles are intended for single‐use only, studies estimate that at least a third of patients reuse their needles, with single‐use needles used up to 19 times or more [3, 4].

Motivations for needle reuse are multifaceted, including economic costs, needle‐related anxiety, dexterity and vision issues, and convenience [5]. However, customer complaint databases recognize needle reuse as the primary, underlying cause of most patient compliance issues: local pain and bruising, needle clogging, and injection site reactions (ISR). Moreover, needle reuse is associated with complications such as lipodystrophy, infections, and compromised glycemic control [6]. While findings are inconsistent, evidence suggests that repeated injections with a damaged/dulled needle may increase tissue trauma and cutaneous complications [3]. Thus, this controversial practice may be unsafe from a biomechanical perspective. However, the research becomes ambiguous regarding the microbial role in these complications and the subsequent risk of infection. Multiple studies have attempted to determine this risk, yet data regarding microbial contamination of pen‐injectors are unclear and undecided [4, 7]. Furthermore, the extent to which needle reuse may cause local skin infections remains unsubstantiated, as no robust data can establish neither correlation nor causality between these rates [3]. Given the notable disparity between the advice of medical authorities and the widespread practice of needle reuse, the discernible lack of documented infections warrants closer inspection [3, 7].

Diabetes is associated with perturbations in skin homeostasis caused by reduced vascularization, neuropathy, and metabolic alterations [8]. The etiopathogenesis involves increased pH, stratum corneum dehydration, and accumulation of advanced glycation end products (AGEs) [8, 9]. Moreover, the hyperglycemic state negatively disturbs the body's responsiveness to antimicrobial therapy while compromising its natural defense against otherwise commensal bacteria [10]. Prior investigations exploring cutaneous dysbiosis in diabetes found significant variations in species diversity [9, 11]. While skin microbiota of regions with similar dermatology has been assessed, the microbiota around injection sites of diabetics is largely unexplored [11, 12]. Moreover, data concerning contamination rates of pen‐injectors remain lacking or inconclusive. Studies have documented biological contamination of drug cartridges due to backflow pressure, indicating potential for transmitting various pathogens [13, 14]. Yet, these papers inspect the drug cartridge only, testing for blood or cells rather than microorganisms. In turn, studies assessing needle contamination are few, outdated, and performed mostly on syringes or older generations of pen‐injectors [15, 16, 17].

This study endeavored to establish the overall risk of microbiological contamination of insulin pens during intended and repeated use. To facilitate a deeper understanding, we first characterized skin microbiota biogeography at injection sites for persons with diabetes that might adhere to pen‐injectors during subcutaneous insulin injections. Microbiological samples were acquired through skin tape strips and skin swabs and evaluated through culture, 16S/ITS high throughput sequencing (HTS), and confocal scanning laser microscopy (CSLM) and compared with non‐diabetic controls. Secondly, we explored the rate and nature of microbial contamination of the needle and cartridge through molecular analysis of used devices. Finally, we constructed a mathematical model to simulate device contamination during subcutaneous injections based on the experimentally obtained data.

## METHODS

### Ethical considerations and study design

Subjects with diabetes were recruited from Steno Diabetes Center Copenhagen, Gentofte Hospital, and Steno Diabetes Center NordJylland, Aalborg University Hospital, Denmark, and controls were recruited through a Danish volunteer recruitment website (forsoegsperson.dk) and by word‐of‐mouth. Data collection was officially exempted from ethical approval by the National Committee on Health Research Ethics case‐nr.: H‐19070331.

Based on previous data, at least 20 subjects per group were required for a power of 0.8 and p < 0.05 statistical significance [18]. Two groups (A and B) of 50 participants with type 2 diabetes and controls matched by age and sex (±3 years) of legally competent age were included. Exclusion criteria included antibiotics use 2 months prior to enrolment, current infections, skin disorders such as psoriasis, and/or wounds in sampling area. Approval and written informed consent were obtained, and metadata was subsequently self‐reported in a pre‐sampling interview.

### Participants

Two groups (group A and B) of 25 participants with type 2 diabetes (n = 50) and corresponding groups of age‐ and sex‐matched (±3 years) control subjects (n = 50) were included in March–June and in November 2020, respectively (total n = 100). Group A consisted of 28 males and 22 females with ages 63.7 ± 11.5 and BMIs 31.0 ± 6.0 for the participants with diabetes ages 63.2 ± 11.08 and BMI 25.0 ± 3.7 of patients without diabetes (controls). Group B consisted of 22 males and 28 females pairwise from (age 65.65 ± 15.73, BMI 31.5 ± 6.9) and controls (age 64.12 ± 15.38, BMI 25.8 ± 5.5).

### Biological material

Participants were sampled in emblematic subcutaneous injection sites either on the right or left side of the periumbilical abdomen and anterior proximal thigh aseptically by the sampling person to avoid contamination.

### Sampling

***Group A:*** 7 layers of DS100 D'Squame Disc tapes (Monaderm, Monaco) were collected from 25 participants with type 2 diabetes (T2 DM) and 25 participants without diabetes (No DM) applying a D‐square pressure instrument (225 g/cm^2^) (Monaderm) for 15 s. Tape 1 was discarded to remove particles, tape 2 was saved for CLSM, tape 3–6 cultured the same day, and tape 7 saved for 16S/ITS HTS. Three used needles and pen‐injectors were collected from participants with diabetes after *home‐use* and returned by envelope through local postage.

***Group B***: Four tape strips (1a, 2a, 1b, and 2b) were sampled pairwise from another 25 participants with type 2 diabetes (T2 DM) and 25 participants without diabetes (No DM). Tape 1a and 2a were saved for HTS, tape 1b for backup, and tape 2b for CLSM. ESwab™ 480Cs (Copan Diagnostics, Murietta, CA, USA) pre‐moisturized by molecular‐grade water (Lonza AccuGENE Molecular Biology Water, Basel Switzerland) were rubbed 50 times in 5 × 5 cm and saved for HTS.

***Group C***: One to three used needles were collected from ~30 patients with diabetes (DM) administered by a nurse on disinfected skin and saved for HTS.

### Sample preparation

#### Needles and cartridges

Under aseptic conditions, needles were cut at base with pliers, while remaining cartridge liquid was removed with a syringe and saved for HTS.

#### Cultivation

Three milliliter 0.9% NaCl were added to tapes 3–6, then degassed and sonicated for 5 min by ultrasonic bath (Branson 2510, Sigma‐Aldrich, St. Louis, MO, USA). Samples were plated in dilution series on 5% blood agar, chocolate agar, and fastidious anaerobic agar (SSI Diagnostica, Hillerød, Denmark) and incubated in aerobic, with CO_2_ (CO_2_ incubator, Sanyo, Osaka Japan) and anaerobic conditions (Concept 400, LAF technologies, Bayswater North, Vic., Australia) at 37°C for 1–3 days. Colonies were quantified in colony forming units (CFU), isolated, and stored at −80°C.

#### MALDI‐TOF identification

Re‐cultivated bacterial isolates were transferred to the MSP 96 target polished steel BC (Bruker, Bremen, Germany), identified with MTB Compass program and MALDI‐TOF microflex LT/SH (Bruker). Reads with scores >2.0 were accepted as identified on species‐level.

#### CLSM

Tapes were stained for 15 min with 3 mg DAPI, placed and sealed onto a microscope slide using clear nail polish and examined using an Axio Imager.Z2, LSM710 CLSM (Zeiss, Oberkochen, Germany) with Plan‐Neoflour and 63×/1.4 plan‐apochromatic oil objectives (Zeiss) and UV light of excitation of 405 nm and emission 410–483 nm. Three representative images per tape were taken using the ZEN black 2010, v. 6.0 (Zeiss) and deconvoluted using Imaris 8 × 64 version 8.1.2 (Bitplane, Zürich, Switzerland).

#### HTS

Sequencing was performed at Clinical Microbiomics A/S, Denmark. RNA was prepared using NucleoSpin® 96 Soil (Macherey‐Nagel, Düren, Nordrhein‐Westfalen, Germany) manual with positive (ZymoBIOMICSTM Microbial Community Standard, Zymo Research, Irvine, CA, USA) and negative controls included with each batch of samples. For PCR, universal primers 341‐F and 785‐R ITS3‐F and ITS4‐R targeted the 16S rDNA and ITS. Indices were added with 8 cycles of 55°C annealing program using the Nextera Index Kit V2 (Illumina, San Diego, CA, USA) and verified on agarose gels. Sequencing was done on an Illumina MiSeq desktop sequencer using the MiSeq Reagent Kit V3 (Illumina) for 2× 300 bp paired‐end sequencing. For bioinformatics analysis, an adjusted DADA2 pipeline was used, primer sequences removed using cutadapt, filtered, and trimmed (dada2::filterAndTrim command) at 3′‐ends on sample‐specific quality. The remaining reads were dereplicated and denoised, forward and reverse reads merged, and pairs without sufficient overlap or mismatch discarded. Default taxonomic using a naïve Bayesian classifier algorithm compared reference databases for ASVs. In silico extracted amplicons were checked against corresponding primers from reference databases [19, 20, 21, 22]. ASVs not present in ≥2 samples or relative abundance less than 0.8% were removed. For fungal sequences, the UNITE ITS database was used [23].

### Mathematical modeling of SCIs

A 2D stochastic mathematical model was constructed to simulate microbial distribution and contamination rates during SCIs. Initially, we tested the assumption of independent, homogenously dispersed colonies with a random, uniform distribution given by:

fx=−1b−a:a≤x≥b,0:x<aorx>b

where *a* and *b* represent boundaries of the area giving a total of 100 mm^2^ (1 cm^2^).

However, CLSM images, previous observations, and studies of cutaneous microbiota in wound studies suggest a heterogeneously dispersed growth in aggregates [24]. A model was implemented, emulating injections through microflora with randomly varying aggregate sizes following a normal distribution given by:

$$f\left(x\right)=\frac{1}{\sigma \sqrt{2}\pi }e^{-\frac{1}{2}\;{\left(\frac{x-\mu }{\sigma }\right)}^{2}},$$

where *μ* is the mean of the distribution, *σ* is its standard deviation, and *e* is Euler's number. Colony size was set to 50 with the standard deviation varying from 0.01 to 0.5 based on microscopic observations and approximations. Finally, research and our CFU determinations suggest that bacteria are dispersed following a Poisson distribution [25]. Assuming that each cluster is independent and random, the distribution is given by:

$$f\left(x,\lambda \right)=\frac{\lambda^{x}e^{-\lambda }}{x!}$$

where *x* is the number of occurrences, and *λ* is the intensity or the expected occurrence of an event per interval (CFU/cm^2^).

For simplification purposes, the needle was modeled as a cylinder, with the tip shaped as a circle given an area by: *A* = *πr* ^2^. The bacteria were quantified as the number positioned either on or within the circle circumference or needle lumen, given by:

$${\left(\frac{x-x_{c}}{r}\right)}^{2}+{\left(\frac{y-y_{c}}{r}\right)}^{2}\leq 1$$

where (*x*, *y*) represents the colony center, (*x*_*c*_, *y*_*c*_) the needle center and *r* the needle radius. The injection was simulated 100 times with 30G (outer diameter (OD) of 0.312 mm) and 32G (OD of 0.235 mm) for each dispersal models with average of 350 CFU/cm^2^, where bacteria penetrated in each simulation was quantified. This was further challenged by the maximum density found from experimental data of 5200 CFU/cm^2^. Algorithm implementation and simulations of SCIs were performed in Python3 (Python Software Foundation, Wilmington, DE, USA). The code can be found in Supporting Information.

### Statistical analyses

All statistical analyses were performed within the computing environment R (v. 3.5.0; R Core Development Team, Boston, MA, USA).

## RESULTS

### Conventional culture

**Fig. 1:**
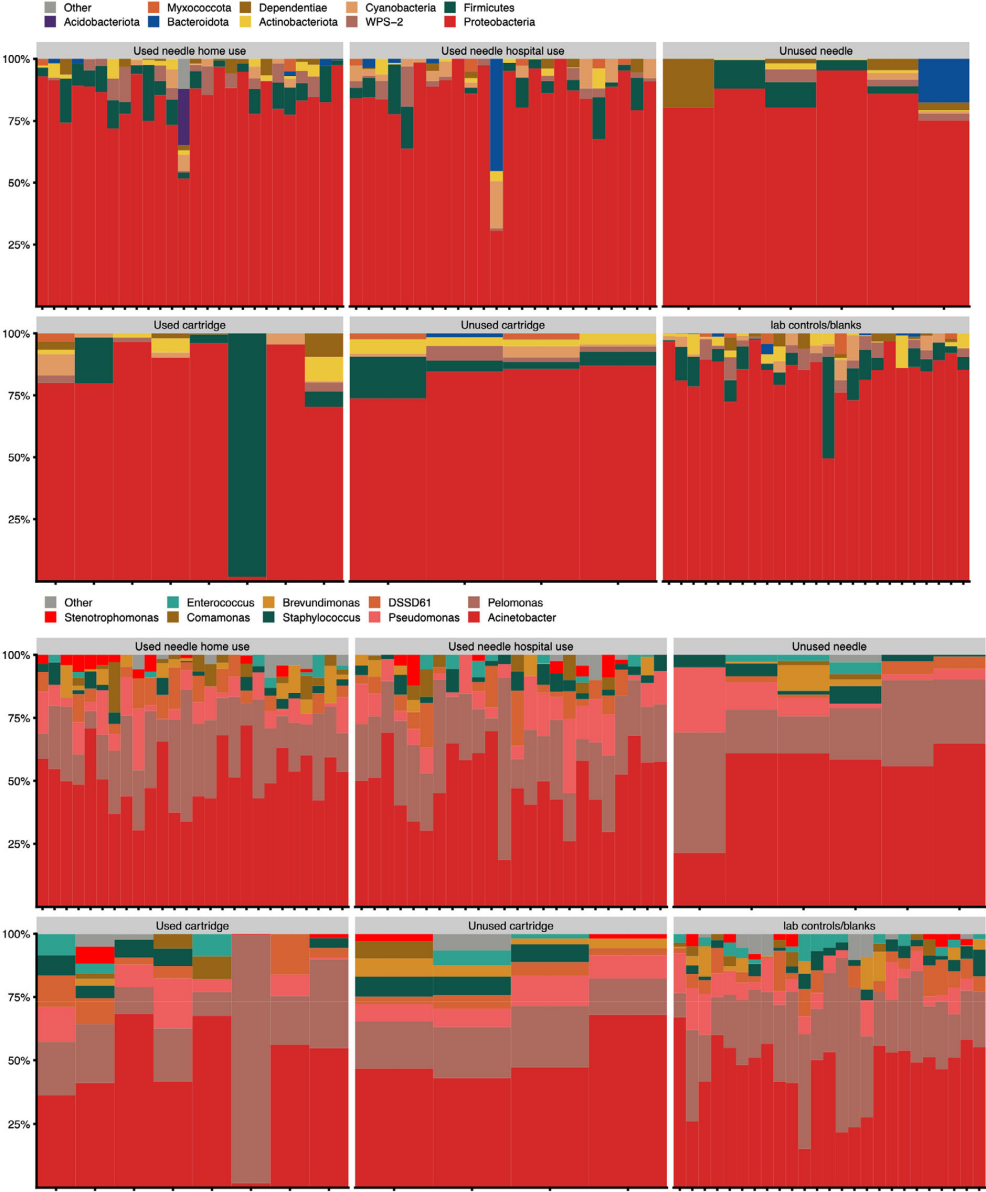
Taxonomic profiles of all samples that passed the described biological signal filter at phylum level. The majority of used devices were dominated by Proteobacteria, similar to the negative controls. This indicates that little biological signal was amplified for those samples.

**Fig. 2:**
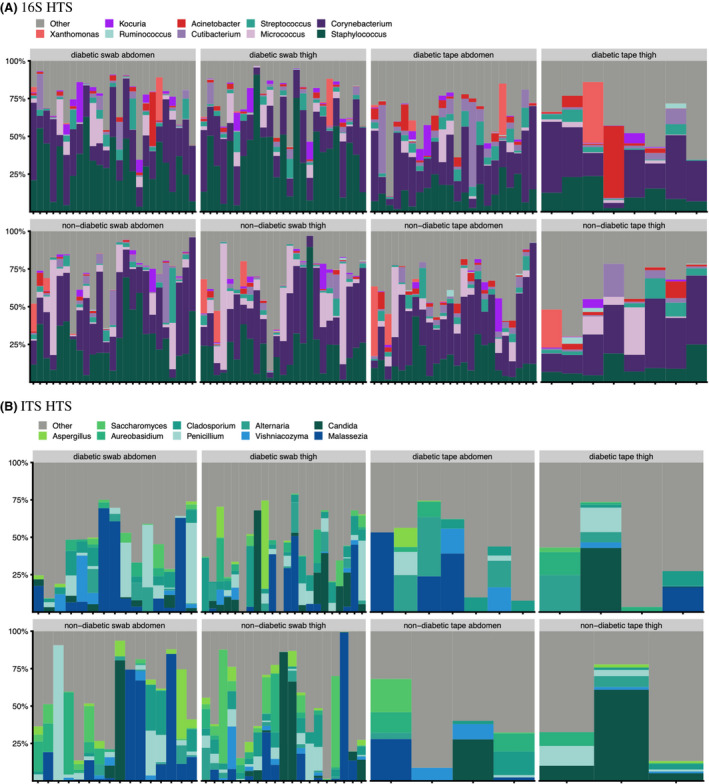
Taxonomic profiles of all diabetic subjects and healthy volunteers with more than 3500 HQ sequences after background removal at genus level for (A) 16S HTS and (B) ITS HTS *staphylococcus* and *Corynebacterium* were the most abundant genera for 16S, while *Candida* and *Malassezia* were the most abundant for ITS. (A) 16S HTS (B) ITS HTS.

In epidermal skin of persons with diabetes, an average of 3·10^2^ and 4·10^2^ CFU/cm^2^ were found on the abdomen and thigh by aerobic and CO_2_ growth conditions, and 2·10^2^ and 4·10^2^ CFU/cm^2^ under anaerobic growth conditions, for abdomen and thigh, respectively. In healthy skin, 3·10^2^ and 2·10^2^ CFU/cm^2^ were found by aerobic and CO_2_ growth, 7·10^1^ and 3·10^1^ CFU/cm^2^ under anaerobic conditions from both areas. The data followed a Poisson distribution, ranging from 0 to 5200 CFU/cm^2^ (Fig. 1 and Table 1 in Supporting Information) and was log‐transformed before a one‐way ANOVA analysis was performed. The ANOVA analysis revealed a greater number of (p = 0.05) viable bacteria on skin from participants with diabetes. Participants with diabetes had significantly (p = 0.05) more anaerobic bacteria, as well as more (p = 0.05) bacteria on thigh compared with controls (Fig. 2).

### MALDI‐TOF

**Fig. 3:**
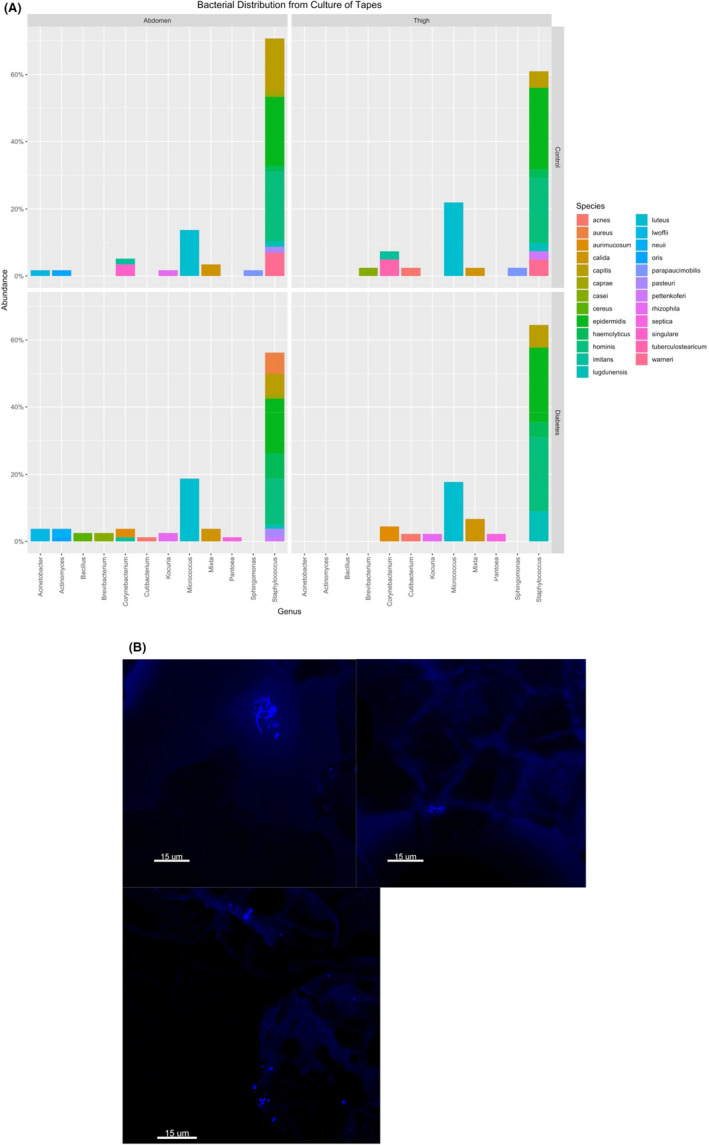
Microbiota in superficial diabetic and healthy skin: (A) viable bacterial species recovered from cultivation of tape strips from abdomen and thigh of participants with diabetes (n = 25) and participants without diabetes (N = 25). (B) Distribution of bacteria on DAPI stained tape strips. Images were taken with Axio Imager.Z2, LSM710 CLSM (Zeiss, Germany) with plan‐Neoflour and 63×/1.4 plan‐apochromatic oil objectives (Zeiss) and UV light of excitation of 405 nm and emission 410–483 nm. Bacteria were heterogeneously distributed in small aggregates or single or diplo scattered cells in the outer stratum corneum.

The down‐stream analysis was performed on species present in ≥2 participants. The outcome included 24 unique species from 12 different genera. Of these, 63.5% were Firmicutes, 29.1% Actinobacteria, and 7.8% Proteobacteria. *Staphylococcus* spp. dominated (61.7%) with *S. epidermidis*, *S. hominis*, and *S. capitis* prevailing. Of all identified, 10.2% were gram‐negative (mostly *Mixta calida* and *Acinetobacter lwoffii*). An overview is shown in Fig. 3A.

### Confocal microscopy

Bacteria were generally scarcely populated, heterogeneous distributed in small aggregates or single or diplo scattered cells. They were found in the outermost stratum corneum as seen in Fig. 3B.

### 16S and ITS high throughput sequencing

From the first round of sampling (group A), only 67/100 and 46/100 of tape 7 from thigh and abdomen surpassed the 3500‐high quality (HQ) reads threshold for composition analyses for 16S and ITS, respectively. Most tapes were dominated by *Proteobacteria* spp., as found in negative controls.

In Group B, 62/68 (91.2%) of tapes and all swabs surpassed the threshold for 16S analysis. Samples were dominated by Actinobacteriota (42.7%, 46.3%), Firmicutes (46.7%, 44.0%), Proteobacteria (8.8%, 7.8%), and Bacteriodita (0.9%, 1.0%) for participants with diabetes and without diabetes, respectively. *Staphylococcus* spp., *Corynebacterium* spp., and *Micrococcus* spp. were the most abundant genera. Generally, intra‐individual taxonomical similarities were found. In swab samples, *Haemophilus* spp. and *Actinomyces* spp. had lower but not significant average abundances in participants with diabetes than without diabetes. The abundance of *S. aureus* showed higher prevalence on abdomen swabs of participants with diabetes (p < =0.05). In addition, *S. aureus* and *S. epidermidis* were more abundant in swab of men samples.

For ITS, 18/68 tapes and 72/100 swabs surpassed the threshold. Samples were dominated by Ascomycota, Basidiomycota, and Mucoromycota with most abundant genera including *Candida* spp., *Malassezia* spp., *Alternaria* spp., and *Vishniacozyma* spp.

A significant effect of sex (p = 0.045) and diabetic status (p = 0.008) was found on swab thigh samples' overall composition, but not on swab or tape abdomen samples. No discernible difference was seen in richness or Shannon diversity index. For diabetic status, no significant taxa were identified.

For ITS, beta diversity analyses showed no significant effect of diabetic status on the overall composition. Comparing alpha diversity values showed mildly increased richness values of swab thigh samples from participants without diabetes than with diabetes, but no differences in Shannon index. Similarly, no alpha diversity differences were seen between sexes. In diabetic samples, *Vishniacozyma* spp. and *Alternaria* were more but not significantly abundant. There were no differentially prevalent taxa between sexes or sampling area. For ITS, sampling area was not significant, though the thigh contained greater abundance of *Candida* spp. and prevalence of *Cladosporium* spp.

A PERMANOVA test showed significant differences in overall composition between swab and tape samples (p = 0.001), explaining 5% of the overall variation. Regarding alpha diversity, swab samples had higher richness, while there were no significant differences in Shannon index values compared with tapes. The most differentially abundant genus was *Staphylococcus* spp., followed by *Corynebacterium* spp., with higher abundances in swabs than tapes. No significant difference was found using ITS.

The majority (93–97% and 86%) of needles and cartridges exhibited no biological signal. Subsequently, 22 species in 26 genera remained (Supporting Information). Genera previously published as DNA contaminants in laboratory consumables were removed, leaving a signal from *Oceanobacillus* spp. Furthermore, the tested devices' origin and corresponding use regimen (single‐use *versus* reuse) had no significant effect on contamination rates.

### Mathematical model

**Fig. 4:**
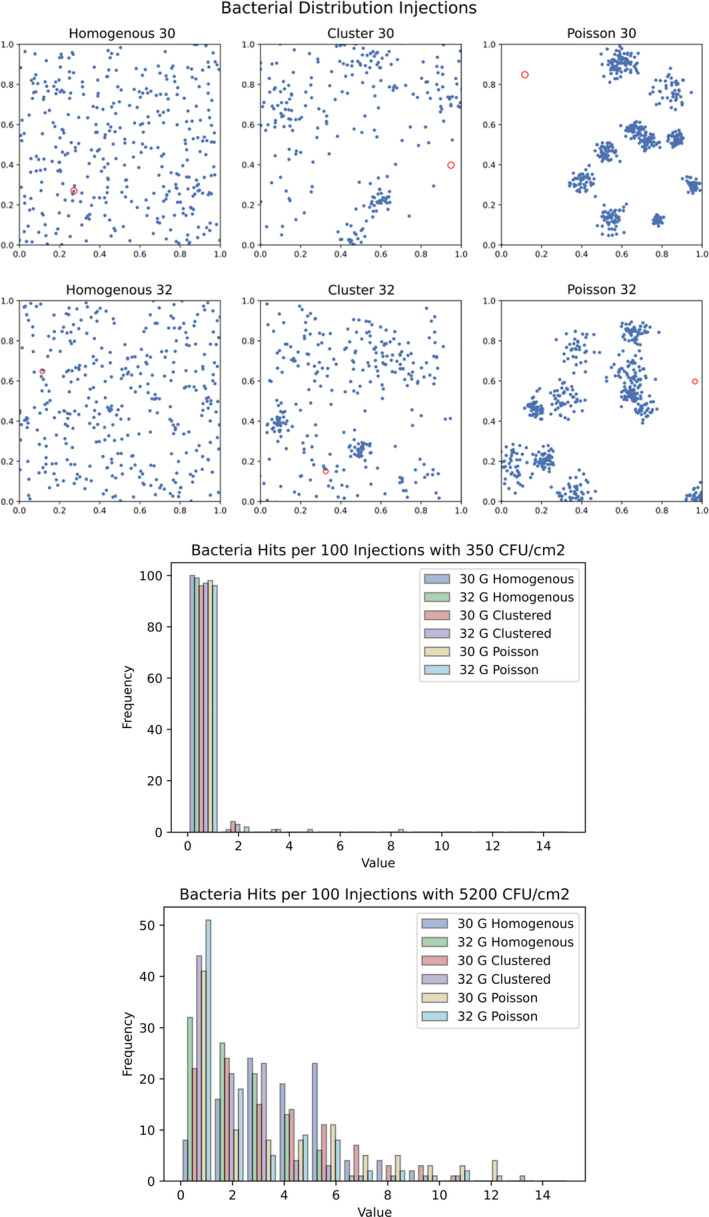
Mathematical model simulating a subcutaneous insulin injection with skin microflora distributed in homogenous, clustered and Poisson dispersal patterns, with needle gauges 30G and 32G and population densities of 350 and 5200 CFU/cm^2^.

Simulations of the subcutaneous injections confirmed the improbability of penetrating a bacterial colony with a needle. As shown in Fig. 4A, the bacterial dispersal renders needle penetration of a large colony of bacteria highly unlikely for all three dispersal patterns and investigated needle gauges. As seen in the histogram of Fig. 4B, a population density of 350 CFU/cm^2^ and homogenous distribution on average penetrated 0.24 ± 0.51 and 0.17 ± 0.38 bacteria with a 30 and 32G needle, respectively. With a clustered dispersal pattern, 0.18 ± 0.49 and 0.11 ± 0.34 were pierced. With the Poisson distribution, 0.19 ± 0.66 and 0.07 ± 0.38 bacteria were displaced. The 32G needle and simulations using a Poisson distribution showed lower average bacteria hit per injection, reflecting the reduced cross‐sectional area of the needle lumen. The simulation repeated with the upper limit (5200 CFU/cm^2^) can be seen in Fig. 4C. Even at this furthermost and excessive scenario, most penetrations encountered no bacteria. Of note, an increase in bacterial population does not correspond to linearly proportional rises in bacteria penetrated, as distribution and needle size were dominating parameters.

## DISCUSSION

Generally, used pen‐injector needles and cartridges exhibited strikingly low amounts of biological signal. In fact, most devices contained insufficient signal for reliable microbiome analyses. Similarly, no fungi were detected in most used needles and cartridges. A fraction of devices revealed low quantities of microbial residue. Genera previously published as DNA contaminants in laboratory consumables were thus removed to circumvent the documented caveat of sequencing low microbial biomass samples [26]. The remaining signal came from the genus *Oceanobacillus*, a halophilic bacteria isolated in multiple environments. Whether this contamination originates from the use or production remains unknown. However, it is generally acknowledged that biological material such as cells and blood can enter the cartridge during injections due to backflow pressure [14]. Nevertheless, drug preservatives such as metacresol and phenol added to stabilize the drug concurrently prevent microbial growth. Consequently, any microorganisms potentially contaminating the drug cartridge will be low in numbers and either killed or deactivated. As such, the clinical implications of reinjecting such bacteria are inconsequential and should be emphasized.

Our CFU determination and microscopy imaging of injection sites reveal a scarce and heterogeneously scattered microflora. Undeniably, simulations through our mathematical model offers vivid testimony to the high improbability of penetrating a bacterial colony with a needle. Even when the model is challenged at the utmost extremes with the uppermost observed bacterial density (5200 CFU/cm^2^) and 100 repeated injections, most needles encounter no bacteria. This is especially of note considering the model's broad oversimplification of the injection process, where bacteria were modeled approximately 300–600× larger than reality (300 μm vs. 0.5–1 μm for visualization purposes). This vast exaggeration renders our findings highly conservative and generously biased toward contamination, as the likelihood of penetrating a bacteria would be lower with greater distances between microbial clusters.

These findings corroborate why no convincing studies document diabetic infections due to subcutaneous injections [3, 7, 27]. The desiccated environment of the skin leaves microbial inhabitants sporadically populated in low numbers, scattered in single cells or small aggregates. Furthermore, needles are siliconized to reduce the penetration force during injections, diminishing bacterial adhesion due to smooth, frictionless surfaces, and lack of nutrients [28]. Results from our mathematical model substantiate that the low microbial population dispersed in random clusters render the risk of hitting a colony exceedingly unlikely. Moreover,even if this occurs and a colony of opportunistic bacteria are injected into the skin, the typical number of bacteria needed to initiate infections are orders of magnitudes higher than the needle would encounter [29].

The superficial skin of the abdomen and thigh was dominated by phyla Actinobacteria, Firmicutes, Proteobacteria, and Bacteroidetes, as commonly found on human skin [30]. The composition of phyla was irrespective of sex, diabetic status, or skin location, confirming the stability of the microbial community [31]. Consistent intrapersonal microbiome compositions were displayed, while a pronounced interpersonal variation dominated other moderating factors such as sex and diabetic status. This is consistent with previous findings, revealing high interpersonal variation in community membership and structure [31, 32]. The average number of aerobic bacteria found were notably at the lower end of the reported 10^2^–10^7^ cells/cm^2^ range [33]. Indeed, dry areas are less populated and more prone to diversity, exhibiting the greatest variability over time [34]. Furthermore, the microflora was dominated by *Staphylococcus* spp. and *Corynebacterium* spp., which generally populate human skin [31, 32]. Interpersonal variation was not affected by sampling location, while intrapersonal variation was greater at the thigh.

The significant increase in viable bacteria found on skin from participants with diabetes, particularly on the thigh, is conspicuous. Metabolic alterations such as perturbed thermoregulatory function, adjusted sweat secretion, and increased surface pH may contribute to enriched conditions for microbial growth [35]. Comparably, significantly more anaerobic bacteria in participants with diabetes could suggest that such metabolic shifts foment anaerobic proliferation. This is noteworthy, as anaerobic bacteria are correlated with amplified severity of diabetic foot ulcers [36, 37]. Aerobes and anaerobes coexist in polymicrobial communities with cooperative and possibly, synergistic effects, propagating the complexity, and possibly pathogenicity of the wound [36]. Specifically, the presence of anaerobic bacteria has been shown to correspond with tissue damage such as ischemia, necrosis, gangrene, and odors, though the causality and specific mechanisms involved are far from understood [36]. Nonetheless, a greater population of anaerobic bacteria could expedite tissue damage, thus intensifying wound perseverance. The high within‐participant consistency confirms that both methods collect reliable skin microbiome samples. While these techniques test only superficial skin, similar community membership has been found from swabs, scrapes and biopsies [30]. Though the external environment defines superficial skin microbiome, both swabs and tapes offer rapid, reliable and noninvasive microbial sampling [18, 34].

As such, needle use and reuse is not problematic in terms of microbial contamination. This finding is thoroughly supported by the absence of documented infections in research [3, 7, 27]. In fact, the risk is so minute, that the World Health Organization (WHO) and its Safe Injection Global Network (WHO‐SIGN) updated its guidelines in 2003 advising that disinfection of perceptibly clean skin before subcutaneous injections is ineffective and unnecessary [38]. While this recommendation remains controversial, it underlines the lack of evidence for, and expert accord of negligible infection risks associated with subcutaneous injections. Our findings of low‐ and randomly dispersed populated injection sites, clarify why. Though research has addressed the correlation between reuse and cutaneous complications, rather, the evidence points to a mechanical source. Since modern needles are designed for single‐use only, reuse may damage the needle by bending or hooking and mechanical erosion of the friction‐reducing coating [6]. Injection with a damaged needle amplifies tissue trauma, increasing occurrence of pain and bruising, and permanent scarring. Moreover, injection into scarred or lipodystrophic lesions may induce erratic insulin absorption, compromising delivery accuracy [39]. Similarly, needle reuse potentiates clogging, where insulin proteins oxidize and dry out or precipitate/crystalize which may obstruct the insulin flow, further affecting delivery accuracy. As such, needle reuse remains problematic due to the robustness and mechanical properties of the pen‐injector and cannot be recommended for safe delivery with current design.

### Limitations

When sampling, participants' bathing and hygienic practices were not controlled for. It is, however, documented that body‐washing can temporarily reduce microbial count by an order of magnitude, and momentarily remove pathogens from skin [40]. Furthermore, the included patients were mostly middle‐aged or elderly. While these ages are representative of patients with type 2 diabetes receiving subcutaneous injections of insulin in Denmark, it is acknowledged that age alters skin microbiome, particularly with changes in diversity [41]. The combined effect of diabetes and age, therefore, should be explored. Moreover, this study investigated patients with diabetes type 2, and therefore fails to evaluate the skin microflora of patients with diabetes type 1. Comparing the skin microbiome of participants with type 1, type 2, and gestational diabetes would be an interesting topic for further exploration. Moreover, confounding factors such as resting metabolism and HbA1c levels were not explored and may influence the skin microbiome. In addition, the collection of insulin pens from *home‐use* and mailing them to researchers of leaves ample opportunity for exposure to unaccounted conditions, such as drying of bacteria. Importantly, our study did not differentiate between viable and non‐viable bacteria on the pen‐injectors, which is of relevance if reinjected. While our findings found trivial microbial traces on the examined devices, subsequent research on device reuse should make this distinction to assess the clinical implications of potential reinjection.

In addition, the microscopy rendered visual quantification of bacterial colonies oversimplified and imprecise. Similarly, tape strips only sampled outer layers of the stratum corneum and omitted residing microbes in sweat ducts and hair follicles. As such, more specific staining techniques such as fluorescence in situ hybridization using peptide nucleic acid probes (PNA‐FISH) in biopsies from injection sites would be an exciting avenue for future investigations. In addition, our model assumed a 2D colony structure, penetrated by circular needle shafts in even punctures. However, microscopic observation revealed anisotropic colonies of multiple layers, and hypodermic needle tips have grinded angles to facilitate skin penetration through forces such as cutting, lubricant friction, and elastic stiffness. These forces likely influence the trajectory and subsequent dislocation of microorganisms, and thus, the rate of needle contamination. Finally, our mathematical model ignores environmental dynamics between microorganisms, such as competition for nutrients and inhibitory mechanisms. In addition, the geometries of the needle tip and bacterial colonies were simplified to spherical shapes and represented solely on a 2D axis. Future research should focus on modeling colony shapes through other more fitting models such as a Boolean distribution with anisotropy ratios from experimental data and include needle shape parameters for a more accurate depiction.

## CONCLUSION

This paper aimed to establish and facilitate a deeper, more comprehensive understanding of the risk of microbial contamination during intended and repeated subcutaneous injections. Participants with diabetes exhibit significantly more viable and anaerobic bacteria, suggesting diabetic status is associated with more favorable growth conditions for skin microflora. Injection site analysis through sampling and microscopy exposed scarce and inconsistently distributed microflora dominated by interpersonal variation. Analysis of used pen‐injectors revealed negligible contamination, whether collected from professional administration in a hospital ward or the *“real‐world”* usage of patients' homes. Finally, the mathematical model confirmed the heterogeneous distribution of microorganisms and subsequent high improbability of penetrating a colony during subcutaneous injections.

Our findings of low‐ and randomly populated injection sites explain why there is a distinct lack of evidence for infections from subcutaneous injections. The microbial contamination of pen‐injectors during intended use and reuse is simply inconsequential. However, the reuse of needles cannot be recommended, as current needle designs' physical dimensions lack robustness for multiple injections without increased risk of tissue damage. Therefore, researchers and industry should attempt to develop mechanically durable needles with self‐cleaning mechanisms to offer simpler, safer, more convenient delivery options for patients.

##  

This work was supported by a research grant from the Danish Diabetes Academy, which is funded by the Novo Nordisk Foundation, grant number NNF17S0031406 and the Innovation Fund grant number 9065‐00120B. In addition, the authors would like to thoroughly thank all volunteers, Dr. Dorte Lindqvist Hansen and the Steno Diabetes Center Copenhagen, Steno Diabetes Center Nord Jylland, Christian Andreasen for statistics, Lasse Kvich and Ida Clement Thaarup for general training in the laboratory/microscope, Dinesh Krishnamoorthy for assistance with Python and, finally, the LEO foundation for funding Lene Bay.

## CONFLICTS OF INTERESTS

SWM, VP, and HB are currently employed at NN. TB and LB are paid consultants for NN. No other potential conflicts of interest were reported. Some of this data was previously presented at the online conference *Advanced Treatment and Technologies in Diabetes (ATTD) 2021*.

## AUTHORS' CONTRIBUTIONS

SWM was involved in literature search and writing, figures and tables, and guarantor. SWM, LB, TB, VP, and HB performed study design. SWM and NF was involved in data collection. SWM and LB performed data analysis. SWM, LB, and TB performed data interpretation. SWM, LB, VP, and TB performed editing and approval of final version of manuscript.

## Supporting information


**Appendix S1.** Supplementary Information.
