# Supplementary material for: Injection site microflora in persons with diabetes: why needle reuse is not associated with increased infections?
Source: APMIS. 2022 May 17;130(7):404–16. doi: 10.1111/apm.13230 (PMC9320873; doi:10.1111/apm.13230)
Supplement: Supplementary file 1 — Appendix S1. Supplementary Information. [file APM-130-404-s001.docx]

SUPPLEMENTARY DATA

***Self-reported needle use results***

From Group A, 25 diabetic patients were asked to describe their typical needle use. Most reported using the needle only once (average 1.98 times). However, some patients described reusing the needle more often, with one patient reusing needles > 15 times. No discernible correlation between years of diabetes and needle-reuse was found. Figure is shown in supplementary data.


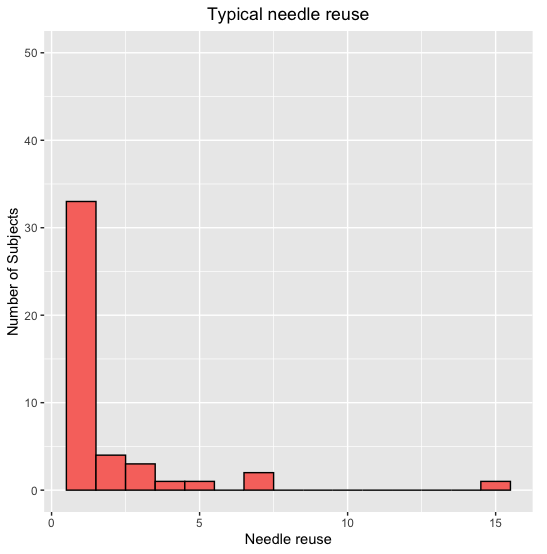

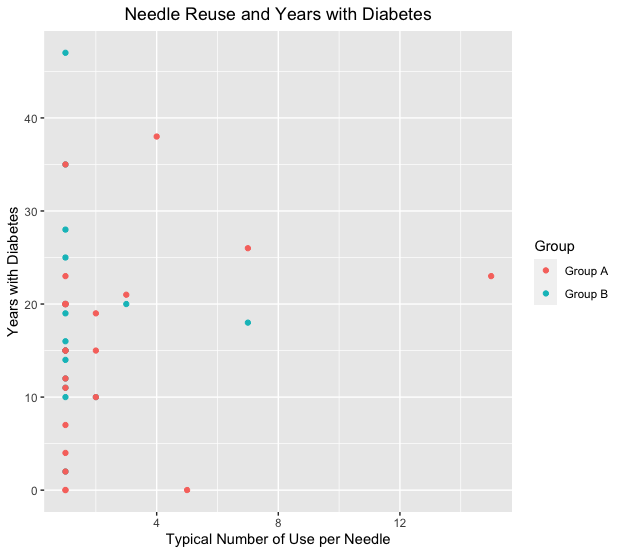


**Figure 1: Distribution of typical needle-reuse surveyed from 50 diabetic patients (T2D) recruited from Steno Diabetes Center Copenhagen (left). Correlation between years with diabetes vs typical needle-reuse from 50 patients (Group A and B) recruited from Steno Diabetes Center Copenhagen. The subjects had on average had diabetes for15.52 years (right). No discernable correlation was seen.**

***Viable bacteria from cultivation***

**Table 1** **Average viable bacteria count from tape 2-5 taken from 50 patients (25 diabetics, 25 control) from abdomen and thigh. The bacteria were cultured in aerobic conditions, with CO_2_, and in anaerobic conditions.**

| **Diabetic status** | **Part** | **Aerobe** | **CO_2_** | **Anaerobe** | **Average** | **Average (Ae+CO_2_)** |
| --- | --- | --- | --- | --- | --- | --- |
| Diabetic | Abdomen | 3.29E+02 | 3.33E+02 | 2.23E+02 | 2.95E+02 | 3.31E+02 |
|  | Thigh | 4.19E+02 | 4.41E+02 | 3.60E+02 | 4.06E+02 | 4.30E+02 |
| Control | Abdomen | 3.12E+02 | 2.13E+02 | 7.16E+01 | 1.99E+02 | 2.62E+02 |
|  | Thigh | 4.00E+02 | 2.43E+02 | 3.12E+01 | 2.25E+02 | 3.22E+02 |
| **Average** |  | 3.65E+02 | 3.07E+02 | 1.72E+02 | 2.81E+02 | 3.36E+02 |


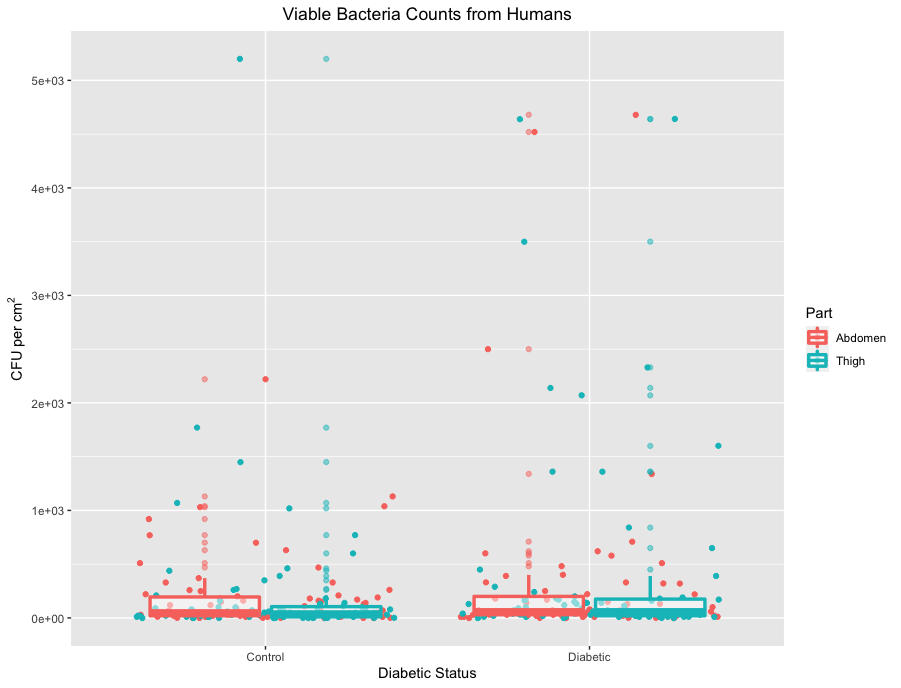


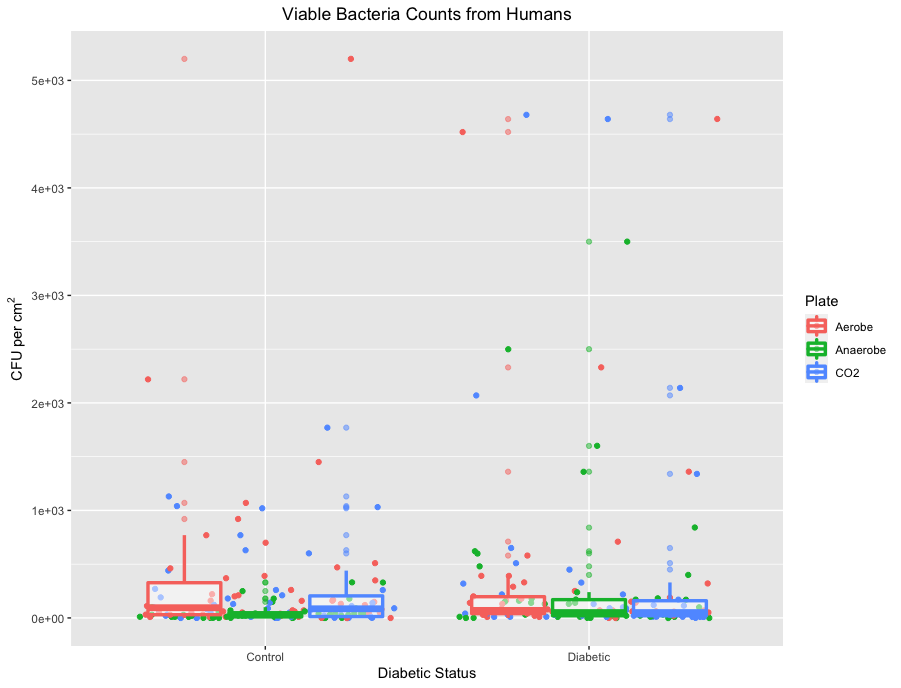


**Figure 2: Distribution of viable bacteria recovered from cultivation of tape strips sampled from group A (25 patients with diabetes (T2 DM) and 25 controls (No DM). The distribution of viable bacteria based on sampling site (abdomen vs. thigh) and incubation condition of cultivation are seen.**

**
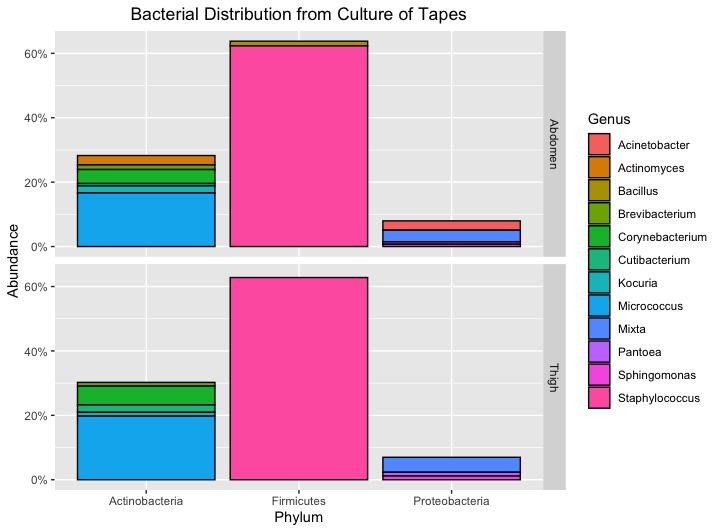
**

**Figure 3: Distribution of viable bacteria recovered from cultivation of tape strips sampled from group A (25 patients with diabetes (T2 DM) and 25 controls (No DM). The distribution of viable bacteria based on sampling site (abdomen vs. thigh) and incubation condition of cultivation are seen.**


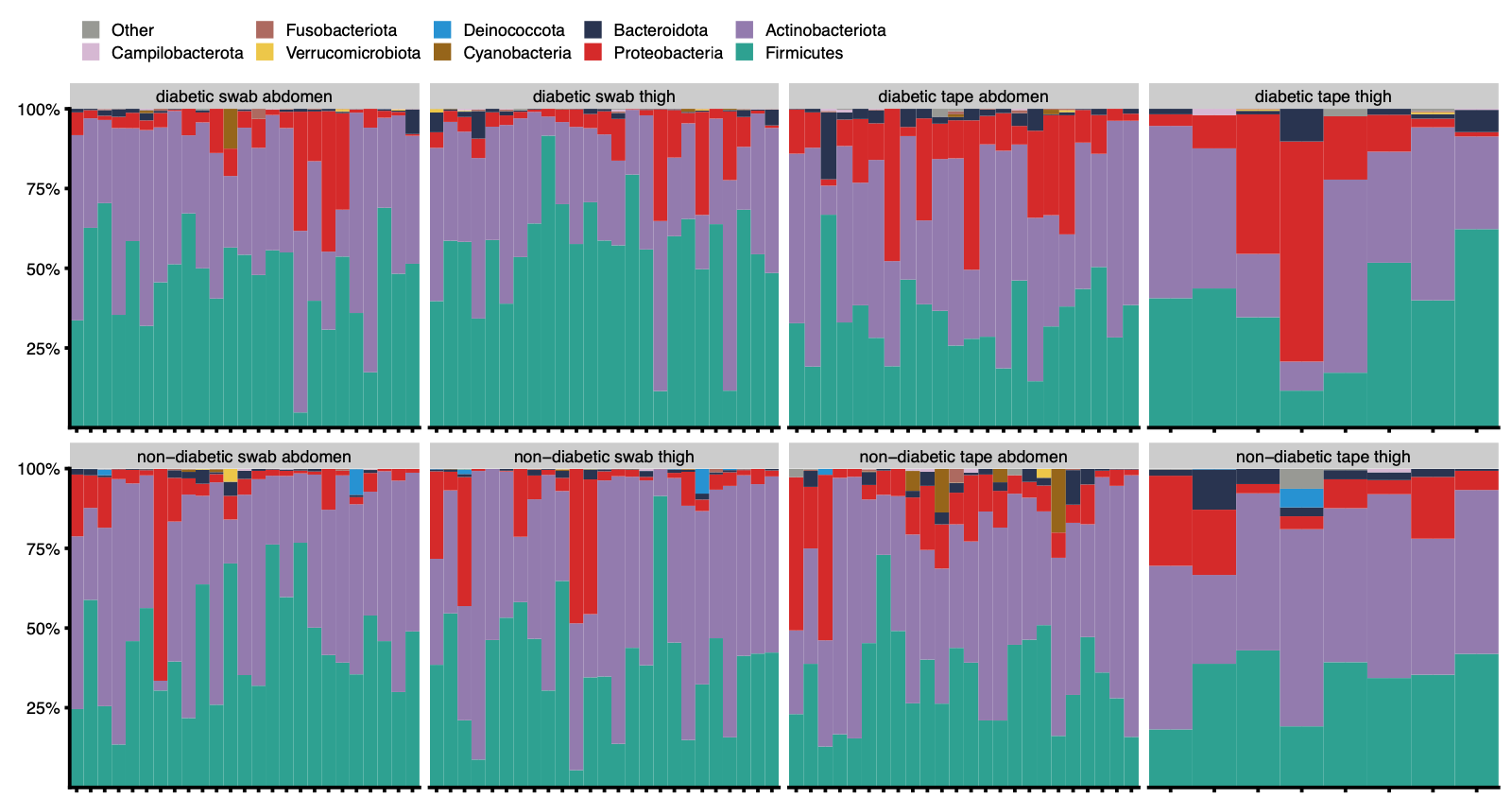


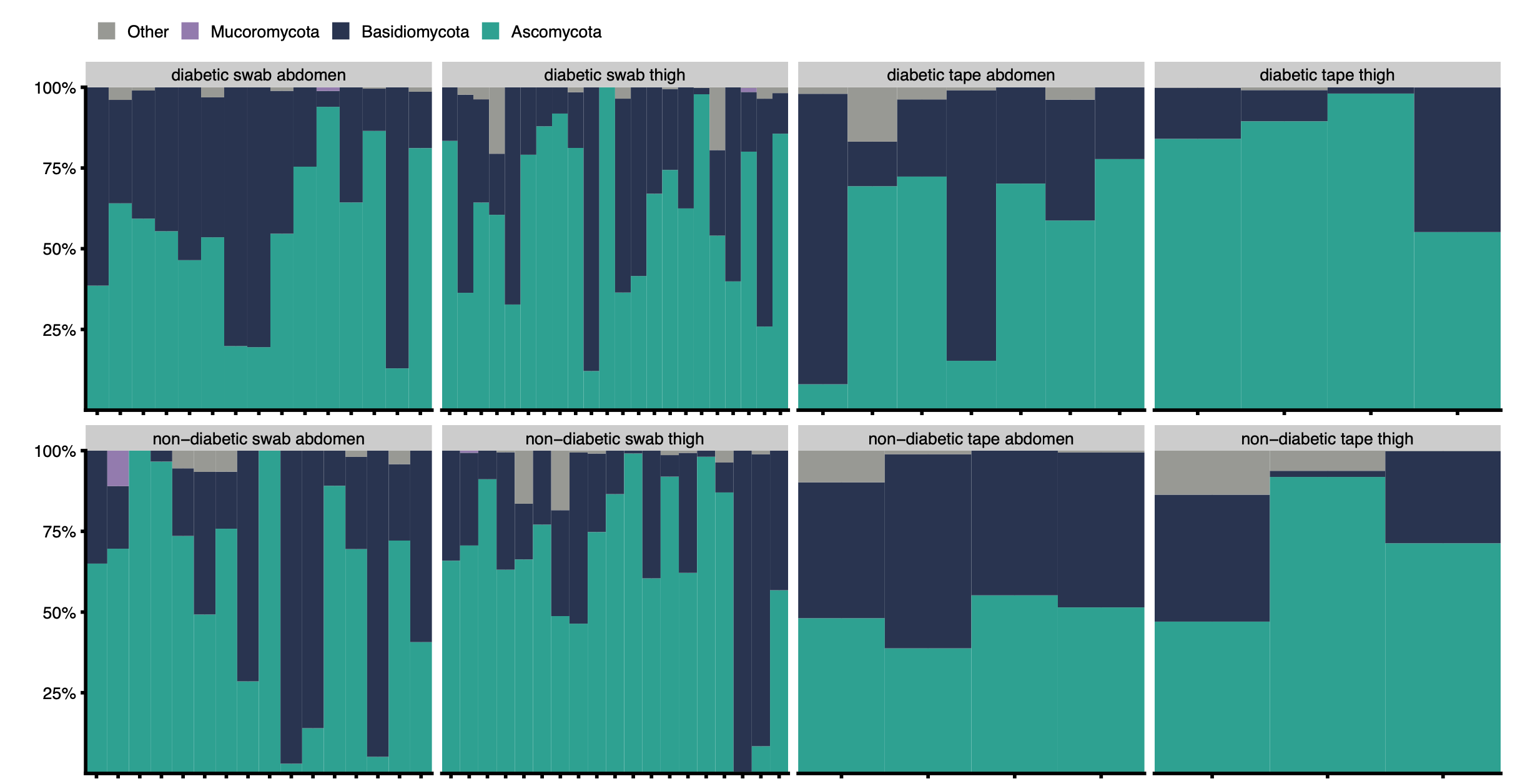
**Figure 4: Taxonomic profiles of all biological samples with more than 3500 HQ sequences after background removal at phylum level (212 samples). All samples are dominated by Firmicutes, Actinobacteriota, and Proteobacteria for 16S and Ascomycota and Basidiomycota for ITS.**

**Table 2** Overview of used pen injectors and needles collected after “in-home use” from patients from Steno Diabetes Center Copenhagen (SCDD) and from the hospital ward at Steno Diabetes Center North Jylland (SDNJ).

| **Device** | **Origin** | **Brand** | **Type** | **Count** |
| --- | --- | --- | --- | --- |
| Needle | SCDD | Klinion | Klinion, 5mm x 0.23 mm (32G) | 5/79 (6.3 %) |
|  |  |  | Klinion, 6 mm x 0.25 mm (31G) | 5/79 (6.3 %) |
|  |  |  | Klinion, Soft fine plus 4 mm x 0.23 mm (32G) | 12/79 (15.2 %) |
|  |  |  | Klinion, Soft fine plus 5 mm x 0,.3 mm (32G) | 2/79 (2.5 %) |
|  |  |  | Klinion, Soft fine plus 6 mm x 0.25 mm (31G) | 16/79 (20.3 %) |
|  |  |  | Klinion, Soft fine plus 8 mm x 0.25 mm (31G) | 9/79 (11.4 %) |
|  |  | Vitrex Medical | Medoject 4 mm x 0.23 mm (32G) | 8/79 (10.1%) |
|  |  | Novo Nordisk | NovoFine 0.36 x 12 mm (28G) | 1/79 (1.3 %) |
|  |  |  | NovoFine Plus 0.25 x 4mm (32G) | 11/79 (13.9 %) |
|  |  |  | NovoTwist 4mm x 0.23 (32 G) | 2/79 (2.5 %) |
|  |  | NA. | Unidentified, 32G | 3/79 (3.8 %) |
|  |  | Verifine | 0.25 x 5mm (31G) | 5/79 (6.3 %) |
|  | SDNJ | Klinion | Klinion, 6mm x 0.25 mm (32G) | 46/46 (100 %) |
| Cartridge | SCDD | Lantus | Lantus Solostar | 7/26 (26.9 %) |
|  |  | Novo Nordisk | NovoRapid | 5/26 (2.5 %) |
|  |  | Toujeo | TeoJeo Solostar | 1/26 (3.8 %) |
|  |  | Novo Nordisk | Tresiba | 9/26 (34.6 %) |
|  |  |  | Victoza | 3/26 (11.5%) |
|  |  |  | Xultrophy | 1/26 (3.8 %) |

***Python code for mathematical model***

﻿#!/usr/bin/env python3

# -*- coding: utf-8 -*-

import numpy as np

import matplotlib.pyplot as plt

from sklearn.datasets import make_blobs

#define cluster distribution

def bacteria_cluster(length = 1, nCFU = 350, col_size = 50, cluster_std_min =0.01, cluster_std_max = 0.5):

# area - skin surface area in cm2

# nCFU - no. of colony forming units per cm2

# col_size - average colony size/ avg no. of bacteria per cluster

area = length**2

total = area*nCFU

centers = int(total/col_size)

X = np.zeros((1,2))

for i in range(0,centers):

cluster_std1 = [np.random.uniform(cluster_std_min,cluster_std_max)]

x,y = make_blobs(n_samples=int(total/centers),centers =int(1) ,n_features=2,

cluster_std=cluster_std1, center_box=(0,length))

X = np.vstack((X,x))

return X

# define uniform distribution

def bacteria_uniform(length = 1, nCFU = 350):

# area - skin surface area in cm2

# nCFU - no. of colony forming units per cm2

area = length**2

total = area*nCFU

x = np.random.uniform(0,length,(total,2))

return x

# define poisson distribution

def bacteria_poisson(length = 1, nCFU = 350, col_size = 50, cluster_std_min =0.01, cluster_std_max = 0.05):

# area - skin surface area in cm2

# nCFU - no. of colony forming units per cm2

# col_size - average colony size/ avg no. of bacteria per cluster

area = length**2

centers = np.random.poisson(area*nCFU/col_size)

numbPoints = np.random.poisson(col_size, centers)

x = np.random.uniform(0,length,centers)

y = np.random.uniform(0,length,centers)

XX = np.zeros((1,1))

YY = np.zeros((1,1))

for i in range(len(numbPoints)):

cluster_std1 = [np.random.uniform(cluster_std_min,cluster_std_max)]

xx = np.random.normal(0, cluster_std1, numbPoints[i]) + x[i]; # x coordinates of Poisson points

yy = np.random.normal(0, cluster_std1, numbPoints[i]) + y[i]; # y coordinates of Poisson points

XX = np.vstack((XX,xx.reshape(-1,1)))

YY = np.vstack((YY,yy.reshape(-1,1)))

return XX,YY,x,y

#define guage

def gauge(x, diameter = 0.23,length=1):

D = diameter

X0,Y0= np.random.uniform(0,length,(2,))

k = 0

for i in range(0,x.shape[0]):

if (x[i,0]-X0)**2 + (x[i,1]-Y0)**2 <= (D/2)**2:

k +=1

return k,(X0,Y0)

#Set gauges

length = 1 #cm skin patch length

D30 = 0.312*0.1 #cm Guage diameter

D32 = 0.235*0.1 #cm Guage diameter

################################

# For g 30 homogenous

N_bac_30_h = []

for i in range(0,100):

x1 =bacteria_uniform(length = length, nCFU = 350)

n_bac,injection_site1 = gauge(x1,diameter =D30,length=length)

N_bac_30_h.append(n_bac)

N_bac_mean_30_h = np.mean(N_bac_30_h)

N_bac_SD_30_h = np.std(N_bac_30_h)

#print(N_bac_30_h)

print(N_bac_mean_30_h, "mean 30 h")

print(N_bac_SD_30_h, "sd 30 h")

injection1 = plt.Circle(tuple(injection_site1),D30/2, color='r',fill=False)

# For g30 cluster

N_bac_30_c = []

for i in range(0,100):

x2 =bacteria_cluster(length = length, nCFU = 350, col_size = 30,cluster_std_min =0.01,cluster_std_max =0.2)

n_bac,injection_site2 = gauge(x2,diameter =D30,length=length)

N_bac_30_c.append(n_bac)

N_bac_mean_30_c = np.mean(N_bac_30_c)

N_bac_SD_30_c = np.std(N_bac_30_c)

#print(N_bac_30_c)

print(N_bac_mean_30_c, "mean 30 c")

print(N_bac_SD_30_c, "sd 30 c")

injection2 = plt.Circle(tuple(injection_site2),D30/2, color='r',fill=False)

# For g32 homogenous

N_bac_32_h = []

for i in range(0,100):

x3 =bacteria_uniform(length = length, nCFU = 350)

n_bac,injection_site3 = gauge(x3,diameter =D32,length=length)

N_bac_32_h.append(n_bac)

N_bac_mean_32_h = np.mean(N_bac_32_h)

N_bac_SD_32_h = np.std(N_bac_32_h)

#print(N_bac_32_h)

print(N_bac_mean_32_h, "mean 32 h")

print(N_bac_SD_32_h, "sd 32 h")

injection3 = plt.Circle(tuple(injection_site3),D32/2, color='r',fill=False)

# For g32 cluster

N_bac_32_c = []

for i in range(0,100):

x4 =bacteria_cluster(length = length, nCFU = 350, col_size = 30, cluster_std_min =0.01,cluster_std_max =0.2)

n_bac,injection_site4 = gauge(x4, diameter =D32,length=length)

N_bac_32_c.append(n_bac)

N_bac_mean_32_c = np.mean(N_bac_32_c)

N_bac_SD_32_c = np.std(N_bac_32_c)

#print(N_bac_32_c)

print(N_bac_mean_32_c, "mean 32 c")

print(N_bac_SD_32_c, "sd 32 c")

injection4 = plt.Circle(tuple(injection_site4),D32/2, color='r',fill=False)

# For g30 poisson

N_bac_30_p = []

for i in range(0,100):

xx,yy,x,y =bacteria_poisson(length = length, nCFU = 350, col_size = 50,cluster_std_min =0.01,cluster_std_max =0.05)

x5 = np.hstack((xx,yy)) #np.array(x5).reshape(-1,2)

n_bac,injection_site5 = gauge(x5, diameter =D30,length=length)

N_bac_30_p.append(n_bac)

N_bac_mean_30_p = np.mean(N_bac_30_p)

N_bac_SD_30_p = np.std(N_bac_30_p)

#print(N_bac_30_p)

print(N_bac_mean_30_p, "mean 30 p")

print(N_bac_SD_30_p, "sd 30 p")

injection5 = plt.Circle(tuple(injection_site5),D30/2, color='r',fill=False)

# For g32 poisson

N_bac_32_p = []

for i in range(0,100):

xx,yy,x,y =bacteria_poisson(length = length, nCFU = 350, col_size = 50,cluster_std_min =0.01,cluster_std_max =0.05)

x6 = np.hstack((xx,yy)) #np.array(x6).reshape(-1,2)

n_bac,injection_site6 = gauge(x6, diameter =D32,length=length)

N_bac_32_p.append(n_bac)

N_bac_mean_32_p = np.mean(N_bac_32_p)

N_bac_SD_32_p = np.std(N_bac_32_p)

#print(N_bac_32_p)

print(N_bac_mean_32_p, "mean 32 p")

print(N_bac_SD_32_p, "sd 32 p")

injection6 = plt.Circle(tuple(injection_site6),D32/2, color='r',fill=False)

# plots

figs, ax = plt.subplots(2,3,figsize=(15,15))

figs.suptitle('Bacterial Distribution Injections' , fontsize=25)

#row 0 column 0

ax[0,0].plot(x1[:,0],x1[:,1],'.')

ax[0,0].add_patch(injection1)

ax[0,0].set_aspect('equal')

ax[0,0].axis([0, 1, 0, 1])

ax[0,0].set_title('Homogenous 30', fontsize=20)

#row 1 column 0

ax[1,0].plot(x3[:,0],x3[:,1],'.')

ax[1,0].add_patch(injection3)

ax[1,0].set_aspect('equal')

ax[1,0].axis([0, 1, 0, 1])

ax[1,0].set_title('Homogenous 32', fontsize=20)

#row 0 column 1

ax[0,1].plot(x2[:,0],x2[:,1],'.')

ax[0,1].add_patch(injection2)

ax[0,1].set_aspect('equal')

ax[0,1].axis([0, 1, 0, 1])

ax[0,1].set_title('Cluster 30', fontsize=20)

#row 1 column 1

ax[1,1].plot(x4[:,0],x4[:,1],'.')

ax[1,1].add_patch(injection4)

ax[1,1].set_aspect('equal')

ax[1,1].axis([0, 1, 0, 1])

ax[1,1].set_title('Cluster 32', fontsize=20)

#row 1 column 2

ax[0,2].plot(x5[:,0],x5[:,1],'.')

ax[0,2].add_patch(injection5)

ax[0,2].set_aspect('equal')

ax[0,2].axis([0, 1, 0, 1])

ax[0,2].set_title('Poisson 30', fontsize=20)

#row 1 column 2

ax[1,2].plot(x6[:,0],x6[:,1],'.')

ax[1,2].add_patch(injection6)

ax[1,2].set_aspect('equal')

ax[1,2].axis([0, 1, 0, 1])

ax[1,2].set_title('Poisson 32', fontsize=20)

figs.savefig('Bactdistreal.svg', format='svg', dpi=1200)

plt.show()

#########

#style of seaborn

plt.style.use('seaborn-deep')

bins = np.linspace(0, 7, 13)

fig2 = plt.hist([N_bac_30_h, N_bac_32_h, N_bac_30_c, N_bac_32_c, N_bac_30_p, N_bac_32_p], bins, alpha=0.5, rwidth= 0.85, edgecolor='black', label=['30 G Homogenous', '32 G Homogenous','30 G Clustered', '32 G Clustered', '32 G Poisson', '32 G Poisson'])

plt.title("Bacteria Hits per 100 Injections, 350 CFU/cm2")

plt.legend(loc='upper right')

plt.xlabel('Value')

plt.ylabel('Frequency')

plt.savefig('Hist350real.svg', format='svg', dpi=1200)

plt.show()
